# *mcr-1* in Carbapenemase-Producing *Klebsiella pneumoniae* with Hospitalized Patients, Portugal, 2016–2017

**DOI:** 10.3201/eid2404.171787

**Published:** 2018-04

**Authors:** Ana Constança Mendes, Ângela Novais, Joana Campos, Carla Rodrigues, Cláudia Santos, Patrícia Antunes, Helena Ramos, Luísa Peixe

**Affiliations:** Centro Hospitalar do Porto, Porto, Portugal (A.C. Mendes, C. Santos, H. Ramos);; Faculdade de Farmácia, Universidade do Porto, Porto (Â. Novais, J. Campos, C. Rodrigues, P. Antunes, L. Peixe);; Faculdade de Ciências da Nutrição e Alimentação, Universidade do Porto, Porto (P. Antunes)

**Keywords:** plasmid-mediated colistin resistance, *mcr*, carbapenem, multidrug resistance, colonization, outbreak, Portugal, *mcr-1*, bacteria, *Klebsiella pneumoniae*, antimicrobial resistance, ST45, IncX4, nosocomial infection

## Abstract

We describe a hospital-based outbreak caused by multidrug-resistant, *Klebsiella pneumoniae* carbapenemase 3–producing, *mcr-1*–positive *K. pneumoniae* sequence type 45 in Portugal. *mcr-1* was located in an IncX4 plasmid. Our data highlight the urgent need for systematic surveillance of *mcr-1* to support adequate therapeutic choices in the nosocomial setting.

Infections with carbapenemase-producing *Enterobacteriaceae* (CPE), such as *Klebsiella pneumoniae*, have been increasing since 2011 in hospitalized patients in several countries in Europe, especially those with high resistance rates (https://ecdc.europa.eu/sites/portal/files/documents/antibiotics-EARS-Net-summary-2016_0.pdf; https://ecdc.europa.eu/sites/portal/files/documents/AMR-surveillance-Europe-2016.pdf). The emergence of mobilized colistin resistance (MCR) genes is particularly concerning because colistin is being intensively used as a last resource antimicrobial drug for treating CPE infections (*1**,**2*). In Europe, sporadic clinical CPE isolates with *mcr-1* have been reported (*3**,**4*). Because CPE has increased at an alarming pace in Portugal (*5**,**6*), we evaluated the occurrence of *mcr-1* among CPE isolated from patients admitted to Centro Hospitalar do Porto, a tertiary and university hospital in Porto, Portugal.

## The Study

**Figure 1:**
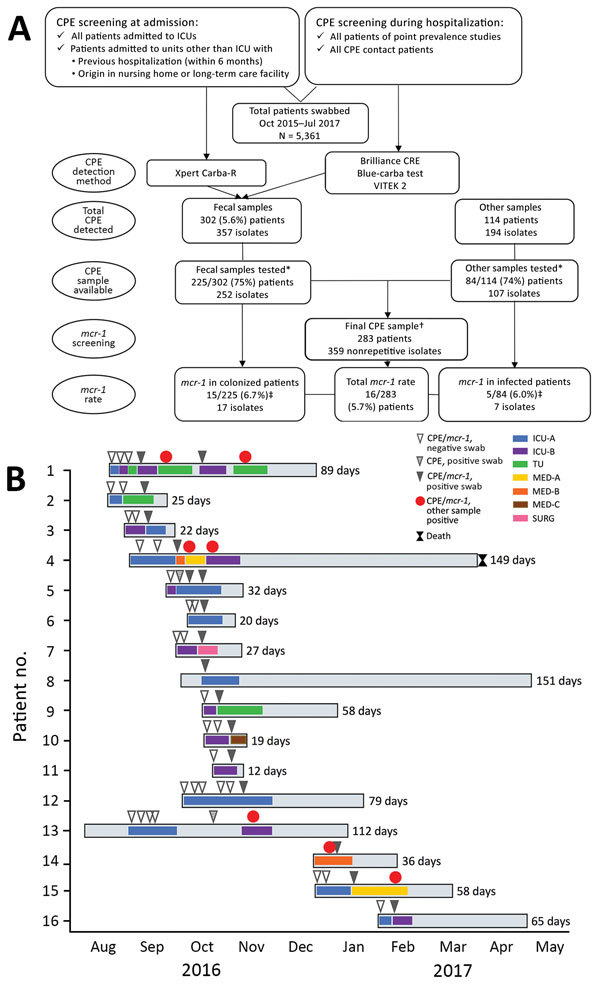
Selection for and testing of patients with *Klebsiella pneumoniae* carbapenemase 3–producing *mcr-1*–positive *Enterobacteriaceae*, Porto, Portugal, 2016–2017. A) Flowchart demonstrating rationale for sample selection. First, we screened for asymptomatic carriage of CPE in the gastrointestinal tract (i.e., colonization by CPE) by testing patient fecal samples with Brilliance CRE Agar (Oxoid, Basingstoke, UK); Xpert Carba-R (Cepheid, Sunnyvale, CA, USA); and VITEK 2 (bioMérieux, Marcy l’Etoile, France). Second, we tested for CPE with all patient samples available. Last, we screened the carbapenemase-producing isolates for *mcr-1* to identify the final sample. *CPE isolates and complete epidemiologic and clinical data were available for ≈75% of CPE patients. †The final sample screened for *mcr-1* included only nonrepetitive isolates. For fecal samples, we considered isolates repetitive when detected in the same patient in samples collected within 72 h from each other. For other types of samples, we considered isolates repetitive when detected in the same sample type collected at the same time point. ‡Four patients carried *mcr-1*–positive isolates either in the gastrointestinal tract or in other body sites. B) Timeline representing epidemiologic data of the 16 patients with *mcr-1*–positive CPE. CPE, carbapenemase-producing *Enterobacteriaceae*; ICU, intensive care unit; MED, medical unit; SURG, surgical unit; TU, transplant unit.

**Figure 2:**
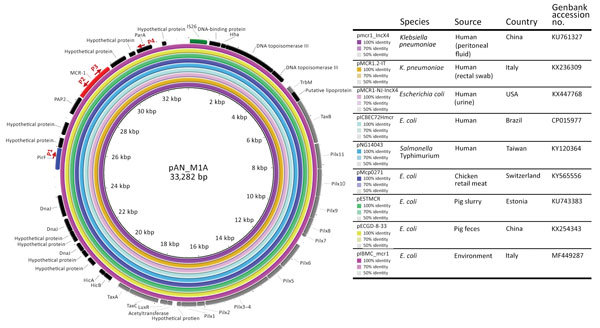
Alignment of representative *mcr-1*–harboring IncX4 plasmids from different isolation sources and geographic regions. The *mcr-1*–harboring plasmid pAN_M1A was used as a reference plasmid. The outermost circle is an annotation of the reference plasmid and shows the direction of transcriptional open-reading frames. The *pil* loci and other genes (gray), replication-associated genes (dark blue), antimicrobial drug resistance gene (red), and insertion sequence (green) are indicated. The strategy for PCR mapping of *mcr-1*–carrying plasmids is indicated by red arrows. Primer P1 targets *pirF*, P2 *mcr-1* (3.3 kb), P3 *mcr-1*, and P4 *parA* (2.1 kb).

Using rectal swab specimens from 5,361 patients admitted to Centro Hospitalar do Porto during October 2015–July 2017, we screened for carbapenemase-positive isolates using Brilliance CRE Agar (Oxoid, Basingstoke, UK), Blue-carba test (*7*), and real-time PCR for carbapenemase genes (Xpert Carba-R; Cepheid, Sunnyvale, CA, USA) (Figure 1, panel A). We identified 283 patients with 359 CPE-positive samples available for further testing. Of the 359 isolates, 252 (75% *K. pneumoniae*–positive) were from patient fecal samples and 107 (86% *K. pneumoniae*-positive) were from other types of patient samples (e.g., blood, urine). We then screened these isolates for *mcr-1*, *bla*_CTX-M-I_-like genes, and *bla*_KPC_ using PCR and sequencing (*5**,**8**,**9*). We determined the antimicrobial drug susceptibility profiles of the *mcr-1*–positive isolates by the broth microdilution method for colistin (http://www.eucast.org/fileadmin/src/media/PDFs/EUCAST_files/General_documents/Recommendations_for_MIC_determination_of_colistin_March_2016.pdf) and by disk diffusion for the other antimicrobial drugs using Clinical and Laboratory Standards Institute/European Committee on Antimicrobial Susceptibility Testing guidelines (http://www.eucast.org/). We evaluated clonal relatedness among *K. pneumoniae* isolates by multilocus sequence and *wzi* capsular typing (http://bigsdb.pasteur.fr/perl/bigsdb/bigsdb.pl?db = pubmlst_klebsiella_seqdef_public) and assessed plasmid replicon content using PCR (*5*). We performed whole-genome sequencing with 2 isolates of the predominant *K. pneumoniae* clones by Hi Seq 2500 Sequencing System (Illumina Inc., San Diego, CA, USA) (2 × 150 bp paired-ended reads, coverage 100×). We assembled reads de novo using SPAdes version 3.9.0 (http://cab.spbu.ru/software/spades/) and annotated contigs with Prokka (http://vicbioinformatics.com/). We used tools from the Center for Genomic Epidemiology (http://www.genomicepidemiology.org) to assess antimicrobial drug resistance genes and replicons and PLACNETw (https://castillo.dicom.unican.es/upload/) for plasmid reconstruction. We located *mcr-1* in the IncX4 plasmid near the replication (*pirF*) and maintenance (*parA*) conserved regions by PCR and sequencing (Figure 2).

We identified 24 carbapenemase-producing and MCR-1–producing *K. pneumoniae* isolates from samples collected during September 2016–February 2017 from 16 hospitalized patients (Figure 1, panel B). Seventeen isolates were colonizers (i.e., bacteria of the patients’ gastrointestinal tract), and 7 were from other parts of the body (3 urine, 2 blood, 2 other biologic fluids) (Table). We recovered 1–4 isolates/patient; 10 colonizing isolates were from intensive care units. Patients (9 men, 7 women) were 50–87 years of age, and their clinical history included prolonged hospitalization (median 47 d, range 12–151 d); complicated conditions; and, for many, surgical interventions, immunosuppression, or previous antimicrobial drug use (usually β-lactams) favoring infection or colonization by multidrug-resistant (MDR) *mcr-1*–positive strains (*10*). Fecal samples were negative for CPE at admission (14/16 patients screened) and for a median of 15 (range 3–94) days after admission (Figure 1, panel B). Five patients had 1 or 2 extraintestinal infections with an MCR-1–producing isolate, sometimes with an isolate identical to one previously detected in their gastrointestinal tract.

**Table:** Demographic and epidemiologic data for 16 patients with *Klebsiella pneumoniae* isolates producing KPC-3 and MCR-1, Porto, Portugal, 2016–2017*

Patient no.	Patient age, y/sex	MLST	Date of isolation	Unit	Specimen type	Antimicrobial drug resistance profile of non-β-lactams†
1	50/M	ST45	2016 Sep 10	ICU-B	Rectal swab	GEN, KAN, NET, TOB, STR, TET, MIN, TGC, FOT, TMP, SXT, NAL, CIP
		ST45	2016 Sep 23	TU	Peritoneal fluid	GEN, KAN, NET, TOB, STR, TET, MIN, TGC, FOT, TMP, SXT, CAM, NAL, CIP
		ST45	2016 Oct 14	ICU-B	Rectal swab	MIN, FOT, TET, TGC, SXT, CAM, NAL, CIP
		ST45	2016 Nov 8	TU	Urine	GEN, KAN, NET, TOB, STR, TET, MIN, TGC, FOT, TMP, SXT, NAL, CIP
2	55/M	ST45	2016 Sep 11	TU	Rectal swab	GEN, NET, TOB, STR, TET, MIN, TGC, FOT, TMP, SXT, CAM, NAL, CIP
3	58/F	ST45	2016 Sep 12	ICU-A	Rectal swab	GEN, KAN, NET, TOB, STR, TET, MIN, TGC, FOT, TMP, SXT, CAM, NAL, CIP
4	73/F	ST45	2016 Oct 1	MED-B	Rectal swab	GEN, KAN, NET, TOB, STR, TET, MIN, TGC, FOT, TMP, SXT, CAM, NAL, CIP
		ST45	2016 Oct 4	MED-A	Urine	GEN, KAN, NET, TOB, STR, TET, MIN, TGC, FOT, TMP, SXT, NAL, CIP
		ST1112	2016 Oct 22	ICU-B	Pus	TET, MIN, FOT, TMP, CAM, NAL, CIP
5	72/M	ST45	2016 Oct 10	ICU-A	Rectal swab	GEN, KAN, NET, TOB, TET, MIN, TGC, FOT, CAM, NAL, CIP
		ST45‡	2016 Oct 14	ICU-A	Rectal swab	GEN, KAN, NET, TOB, TET, MIN, TGC, FOT, CAM, NAL, CIP
6	75/M	ST45	2016 Oct 14	ICU-A	Rectal swab	GEN, KAN, NET, TOB, STR, TET, MIN, TGC, FOT, TMP, SXT, CAM, NAL, CIP
7	68/M	ST45	2016 Oct 14	SURG	Rectal swab	GEN, KAN, TOB, STR, TET, MIN, TGC, FOT, TMP, SXT, NAL, CIP
8	78/F	ST45	2016 Oct 15	ICU-A	Rectal swab	GEN, KAN, NET, TOB, STR, TET, MIN, TGC, FOT, TMP, SXT, CAM, NAL, CIP
9	58/M	ST45	2016 Oct 25	TU	Rectal swab	GEN, KAN, NET, TOB, STR, TET, MIN, TGC, FOT, TMP, SXT, CAM, NAL, CIP
10	51/M	ST45	2016 Nov 1	MED-C	Rectal swab	KAN, NET, TOB, STR, TET, MIN, TGC, TMP, SXT, CAM, NAL, CIP
11	87/F	ST45‡	2016 Nov 1	ICU-B	Rectal swab	GEN, KAN, STR, TET, MIN, TGC, FOT, TMP, SXT, NAL, CIP
12	67/F	ST45	2016 Nov 7	ICU-A	Rectal swab	GEN, TOB, STR, MIN, TGC, FOT, CAM, NAL, CIP
13	57/M	ST45	2016 Nov 7	ICU-B	Blood	GEN, NET, TOB, STR, TET, MIN, TGC, FOT, CAM, NAL
14	76/M	ST45	2016 Dec 30	MED-B	Blood	GEN, KAN, NET, TOB, STR, TET, MIN, TGC, FOT, TMP, SXT, CAM, NAL, CIP
		ST45	2017 Jan 2	MED-B	Rectal swab	GEN, KAN, NET, TOB, STR, TET, MIN, TGC, TMP, SXT, NAL, CIP
15	85/F	ST45	2017 Jan 16	MED-A	Rectal swab	GEN, KAN, NET, TOB, STR, TET, MIN, TGC, FOT, TMP, SXT, CAM, NAL, CIP
		ST45	2017 Feb 7	MED-A	Urine	GEN, NET, TOB, STR, TGC, TMP, SXT, NAL, CIP
16	63/F	ST45	2017 Feb 7	ICU-B	Rectal swab	GEN, KAN, NET, TOB, STR, TET, MIN, TGC, TMP, SXT, NAL, CIP

*CAM, chloramphenicol; CIP, ciprofloxacin; FOT, fosfomycin; GEN, gentamicin; ICU, intensive care unit; KAN, kanamycin; KPC-3, *K. pneumoniae* carbapenemase 3; MCR-1, mobilized colistin resistance 1; MED, medical unit; MIN, minocycline; MLST, multilocus sequence type; NAL, nalidixic acid; NET, netilmicin; SURG, surgical unit; ST, sequence type; STR, streptomycin; SXT, trimethoprim/sulfamethoxazole; TET, tetracycline; TGC, tigecycline; TOB, tobramycin; TMP, trimethoprim; TU, transplant unit. †We considered isolates with intermediate susceptibility profiles resistant. ‡Isolates selected for whole-genome sequencing.

Colistin use and travel abroad were not recorded for any patient before *mcr-1* detection; however, 5 of the 16 patients had been hospitalized in the previous 6 months. Patients were treated for CPE infection with colistin and a carbapenem, which was supplemented with fosfomycin, tigecycline, or piperacillin/tazobactam depending on clinical criteria. We missed colistin resistance initially because we used conventional antimicrobial susceptibility tests VITEK 2 (bioMérieux, Marcy l’Etoile, France) and Etest (bioMérieux), which are unreliable at detecting colistin resistance. Adequate colistin resistance monitoring (http://www.eucast.org/fileadmin/src/media/PDFs/EUCAST_files/General_documents/Recommendations_for_MIC_determination_of_colistin_March_2016.pdf) and *mcr-1* screening for CPE isolates was implemented in July 2017.

Isolates carrying *mcr-1.1* were resistant to colistin (MIC 4–8 mg/L), produced *K. pneumoniae* carbapenemase 3 (KPC-3), and most (79%) produced CTX-M-15 β-lactamase. Besides 100% resistance to third and fourth generation cephalosporins and monobactams, *K. pneumoniae* isolates were also frequently resistant to nalidixic acid (100%), ciprofloxacin (96%), tigecycline (96%), tetracycline (92%), tobramycin (88%), gentamicin (88%), fosfomycin (83%), trimethoprim/sulfamethoxazole (79%), and chloramphenicol (67%) (Table). All isolates were susceptible to amikacin (which was contraindicated for some patients because of renal insufficiency) and ceftazidime/avibactam (which was not available).

All but 1 *K. pneumoniae* isolate belonged to sequence type (ST) 45 and carried *wzi*101/K24, a clone that has been infrequently detected among clinical MDR *K. pneumoniae* isolates in Portugal (*5**,**6*) but has circulated among KPC-3 producers (without *mcr-1*) during the same period (L. Peixe, unpub. data). We detected 1 *mcr-1*–positive *K. pneumoniae* (capsular type KL122) ST1112 isolate from the pus of an abdominal wall abscess in a patient having *mcr-1*–positive ST45 in previously collected fecal and urine samples (Table). The 2 whole-genome–sequenced *K. pneumoniae* ST45 isolates had genes encoding resistance to aminoglycosides [*aac(6’)Ib-cr*,*aac(**3**)-IIa*]; β-lactams (*bla*_KPC-3_, *bla*_SHV-1_, *bla*_OXA-1_), fluoroquinolones [*qnrB66*, *aac(6’)Ib-cr*,*oqxAB*], and other antimicrobial drugs [*catB4*,*tet*(A)]; 1 of the 2 isolates possessed additional genes *aph(**4**)-Ib*, *strAB*, *bla*_TEM-1B_, *bla*_CTX-M-15_, *catA1*, *sul2*, and *dfrA14*.

In all *mcr-1*–positive isolates, the gene was located in an IncX4-type plasmid (Figure 2). Comparative genomics revealed that this plasmid (pAN_M1A) is circulating among diverse hosts (humans, pig, poultry) and the environment in many different countries, including Portugal (*11*). We identified *bla*_KPC-3_ in a Tn*4401d* isoform in an ≈58-kb IncN-ST15 plasmid, a minority platform in our previous survey (*5*); *bla*_CTX-M-15_ was associated with multireplicon plasmid IncFII_K_-FIA-FIB. We deposited this whole-genome shotgun project at DDBJ/European Nucleotide Archive/GenBank under accession no. PEHI00000000.

We found that 5.7% (16/283) of hospitalized patients had gastrointestinal tracts colonized with *mcr-1*–positive CPE, and in 1.8% (5/283) of these patients, an infection developed; these rates are comparable with those reported in China (up to 6.2% for fecal colonization, 1% for infections) (*10*,*12*). In China, only 1 outbreak involving *mcr-1*–carrying clinical isolates has been reported (*13*), and in Europe, a low occurrence (<1%) and sporadic clinical cases have been reported (*3**,**4*). Colistin is a critical last resource antimicrobial drug; prolonged carriage of *mcr-1*–positive MDR strains (especially by patients at discharge) represents a risk for subsequent infections and dissemination to other *Enterobacteriaceae* species. Of note, identifying CPE asymptomatic carriers at discharge is a practice recommended in Portugal, though not mandatory.

Considering the absence of CPE at admission, nosocomial acquisition and in-hospital dissemination of KPC-3–producing strains carrying *mcr-1* is plausible; however, we cannot rule out that other *K. pneumoniae* lineages or *Escherichia coli* might have been the source of *mcr-1*. Although the prevalence of colonization of humans by *mcr-1*–positive strains is unknown in Portugal, previous detection of *mcr-1* in livestock, such as *K. pneumoniae* ST45 in pigs, suggests transmission through the food chain and wider dispersion of MCR-1–producing *Enterobacteriaceae* (*8**,**11**,**14**,**15*).

## Conclusions

We report the emergence of *mcr-1* in MDR KPC-3–producing *K. pneumoniae* associated with an unnoticed outbreak. High rates of CPE and colistin use (*2**,**5**,**6*) together with an ongoing community-based dissemination of *mcr* forebodes future similar events. Our data stress the need for a concerted action involving different professionals and healthcare institutions to monitor and contain the spread of *mcr* across human and veterinary niches, the food chain, and the environment.
